# Popular Treatments of Psoriasis on Social Media: Google Trends Analysis

**DOI:** 10.2196/70067

**Published:** 2025-03-28

**Authors:** Derek Nguyen, Jennifer Javaheri, Ruth Sanchez, Vy Han

**Affiliations:** 1 Psychology Department, University of California, Riverside, 900 University Ave, Riverside, CA, 92521, United States, 1 7148802250; 2 California University of Science and Medicine, Colton, CA, United States

**Keywords:** psoriasis, biologics, Google Trends, Reddit, Facebook, treatment

## Abstract

This study analyzes the most commonly mentioned psoriasis treatments on Facebook and Reddit forums, tracking their popularity over time by using Google Trends.

## Introduction

Approximately 8 million individuals in the United States and 125 million individuals worldwide experience psoriasis—a chronic inflammatory skin disease most commonly characterized by scaly erythematous plaques on the extensor surfaces, face, trunk, and scalp. With increased internet accessibility, many patients now turn to web-based platforms to connect with others and seek advice on managing their condition. Over 30% of internet users report using social media to find health-related information, with forums and web-based communities being among the most popular sources [1]. Notably, two widely used resources in the psoriasis community are the “Psoriasis” group on Facebook and “r/Psoriasis” on Reddit, which collectively have over 110,000 users worldwide. We aimed to analyze the most frequently mentioned treatments on these forums while also exploring how interest has evolved over time. By comparing patient discussions with search trends, this research provides valuable insights into treatment preferences and shifts in public awareness.

## Methods

Using the web application PullPush API—an indexing service that enables users to retrieve content beyond Reddit’s 1000-entry search limit—all posts on both web-based forums from May 23 to November 23, 2024, were compiled and reviewed to assess their relevance to psoriasis treatment, before tallying the number of mentions each unique treatment received. Non-English posts were excluded from data collection due to their small sample size and practical limitations. The 10 most mentioned treatments across both forums were inputted into Google Trends to assess their popularity over time. These treatments were further categorized into groups—disease-modifying antirheumatic drugs (DMARDs), steroids, and procedures. Google Trends allows users to track the popularity of queries by displaying search interest for queries as relative search volumes (RSVs). RSVs range from 1 to 100, where the number indicates how a topic’s search interest compares to its peak interest level. To convert this value to the total number of searches at any given time, the Google Chrome extension Glimpse was used to convert all RSVs to absolute search volumes.

## Results

**Figure 1.**
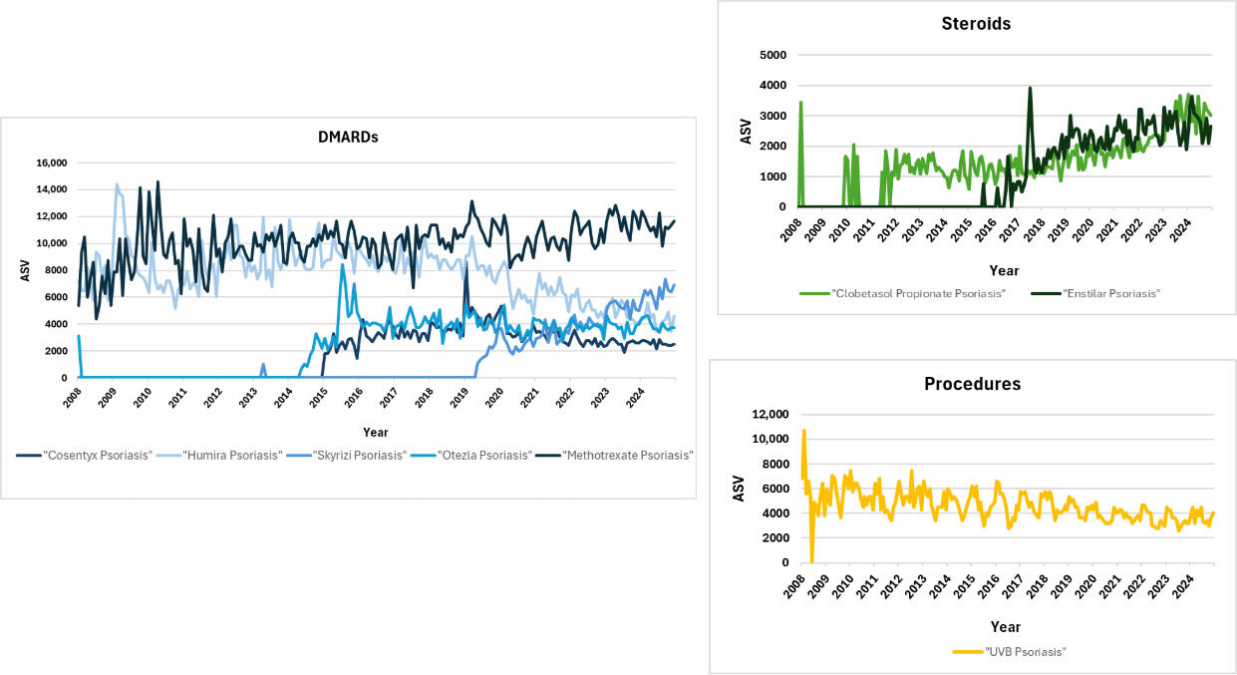
ASV data from Google Trends for the 10 most discussed psoriasis treatments on Reddit and Facebook (2008‐2024). ASV: absolute search volume; DMARD: disease-modifying antirheumatic drug.

The gathered posts contained 2260 unique mentions of treatments, which were categorized into 205 unique treatments. Treatments fell under the following categories: biologics, procedures, dietary modifications, home remedies, and topicals. The 10 most mentioned treatments across both forums are shown in Table 1. Of these, 2 did not meet the minimum search queries necessary to generate graphical data through Google Trends. Among the remaining treatments, those with the highest number of searches in any month between 2008 and 2024 were methotrexate, with 13,860 searches in January 2010, and Humira, with 14,396 searches in February 2009 (Figure 1). These results could be explained by shortages in methotrexate and other oncology drugs between 2010 and 2011 and the US Food and Drug Administration’s approval of Humira for plaque psoriasis in 2008, respectively [2]. In recent years however, Humira has experienced declines in popularity due to the availability of cheaper biosimilars [3]. UV-B phototherapy has likewise experienced a similar trend due to the increase in biologics use [4]. Overall, the subcategories that displayed the highest interest based on the daily number of searches were biologic DMARDs (searches: n=768), steroids (searches: n=93), and procedures (searches: n=151).

**Table 1.** Most mentioned treatments^a^ for psoriasis across Facebook and Reddit in 2024.

Treatment	Mentions (N=2260), n (%)
Risankizumab^b^	181 (8)
UV-B phototherapy^c^	129 (5.7)
Apremilast^b^	103 (4.6)
Methotrexate^b^	102 (4.5)
Adalimumab^b^	97 (4.3)
Guselkumab^b^	81 (3.6)
Secukinumab^b^	72 (3.2)
Clobetasol propionate^d^	70 (3.1)
Cal/BD^e^ foam^d^	68 (3)
Ixekizumab^b^	50 (2.2)
Remaining 195 treatments	1307 (57.8)

a In total, there were 205 unique treatments mentioned.

b Categorized as a disease-modifying antirheumatic drug.

c Categorized as a procedure.

d Categorized as a steroid.

e Cal/BD: calcipotriol/betamethasone dipropionate.

## Discussion

Google Trends data reveal that patients prefer injectable medications, especially biologics, for treating psoriasis, with 5 of the top 10 treatments falling into this category. This trend aligns with previous research highlighting patients’ appreciation for the efficiency and convenience of biologics [5]. However, given past research indicating an increased risk of developing cutaneous disorders, inflammatory bowel disease, or interstitial lung disease, patients should be informed about alternative treatment options [6]. This study’s limitations include the exclusion of other social media platforms and potential selection bias, as social media use tends to be more prevalent among younger demographics [7].

Due to the significant disease burden associated with psoriasis, many patients seek additional treatment options, of which some lack strong evidence. Notably, 51% of patients report using complementary and alternative medicine (CAM), including herbal therapy, climatotherapy, and dietary changes, with many forum users recommending dairy-free or gluten-free diets. Common reasons for these choices include preferences for natural approaches, cultural factors, and a perception that conventional medicine is more toxic than CAM treatments [8,9]. Given their rising popularity, understanding the data surrounding the efficacy of these treatments and their interactions with conventional medicine will better equip dermatologists to serve patients. Interest in psoriasis treatments should center on expanding the evidence base for conventional and alternative treatments and fostering effective collaboration between patients and physicians to optimize outcomes.

## Abbreviations

CAM: complementary and alternative medicine
DMARD: disease-modifying antirheumatic drug
RSV: relative search volume
